# Acoustic Radiation Force Impulse Elastography for Efficacy Evaluation after Hepatocellular Carcinoma Radiofrequency Ablation: A Comparative Study with Contrast-Enhanced Ultrasound

**DOI:** 10.1155/2014/901642

**Published:** 2014-05-06

**Authors:** Xiaohong Xu, Liangping Luo, Jiexin Chen, Jiexin Wang, Honglian Zhou, Mingyi Li, Zhanqiang Jin, Nianping Chen, Huilai Miao, Manzhou Lin, Wei Dai, Anil T. Ahuja, Yi-Xiang J. Wang

**Affiliations:** ^1^The First Affiliated Hospital of Jinan University, Guangzhou 510632, China; ^2^The Affiliated Hospital of Guangdong Medical College, Zhanjiang 524001, China; ^3^Department of Imaging & Interventional Radiology, Prince of Wales Hospital, The Chinese University of Hong Kong, Shatin, N.T., Hong Kong

## Abstract

*Aim*. To explore acoustic radiation force impulse (ARFI) elastography in assessing residual tumors of hepatocellular carcinoma (HCC) after radiofrequency ablation (RFA). *Materials and Methods*. There were 83 HCC lesions among 72 patients. All patients were examined with ARFI, contrast enhanced ultrasound (CEUS), and CT or MRI. Tumor brightness on virtual touch tissue imaging (VTI) and shear wave velocity (SWV) were assessed before and approximately one month after RFA. *Results*. There were 14 residual tumors after RFA. VTI showed that all the tumors were darker after RFA. VTI was not able to distinguish the ablated lesions and the residual tumors. 13 residual tumor lesions were detected by CEUS. All completely ablated nodules had SWV demonstration of x.xx., while with those residual nodules, 6 tumors had x.xx measurement and 8 tumors had measurable SWV. nine lesions with residual tumors occurred in cirrhosis subjects and 5 lesions with residual tumors occurred in fibrosis subjects; there was no residual tumor in the normal liver subjects. *Conclusion*. VTI technique cannot demonstrate residual tumor post RFA. While SWV measurement of less than x.xx is likely associated with residual tumors, measurement of less than x.xx cannot exclude residual tumors. Liver cirrhosis is associated with decreased chance of a complete ablation.

## 1. Introduction

Primary hepatocellular carcinoma (HCC) is one of the most common malignancies. Although surgery demonstrates highest possibility for curing HCC, only 20–30% patients have opportunities of surgical treatment because HCC often occurs on the basis of hepatitis and liver cirrhosis and presents with multifocal lesion [1–3]. Recently radiofrequency ablation (RFA) is developed as one of the popular techniques for tumor ablation [2]. Using resistive ionic heating through electrodes, it can lead to coagulation necrosis of tumor. This technique is commonly used clinically because it is highly effective, minimally invasive, and requires fewer sessions [2]. Several randomized clinical trials have also confirmed that for small HCC, treatment efficacy of thermal ablation is comparable to that of surgical resection [3–5]. However, after RFA residual tumor may still exist [2]. The evaluation of treatment efficacy after percutaneous ablation therapy for HCC is essential for the determination of subsequent treatment and follow-up strategy. Dynamic contrast-enhanced CT and MRI are the standard techniques to evaluate the clinical effectiveness of RFA [6, 7]. Contrast-enhanced Ultrasound (CEUS) is also used to evaluate treatment efficacy after ablation therapy. The agreement between CEUS and contrast-enhanced CT, as well as MRI, has been reported to be good [8].

Acoustic radiation force impulse (ARFI) imaging is a new ultrasound-based diagnostic technique that, evaluating the wave propagation speed, allows the assessment of tissue stiffness [9]. ARFI does not need external compression so the operator dependency is reduced. By short-duration acoustic radiation forces (less than 1 ms), ARFI generates localized displacements in a selected region of interest (ROI) identified on a conventional B-mode image. Depending on the interactions with the transducer, the generated wave scan provides qualitative (imaging) or quantitative (wave velocity values, measured in m/s) responses, by virtual touch tissue imaging (VTI) and virtual touch tissue quantification (VTQ) techniques, respectively (Siemens, Erlangen, Germany) [9]. VTI image is based on the degree of lightness and darkness in the regions of interest for different tissue elasticity coded display of hardness, with the more light color indicating the more soft tissue, the darker color the more hard tissue. VTQ is based on the conventional two-dimensional sonogram with a longitudinal wave emitted by the probe, resulting in a horizontal elastic shear wave propagation in tissue and detect the shear wave velocity to provide information on tissue stiffness, with the more hard tissue showing faster shear wave velocity. There are a number of studies using ARFI in differentiating cancer from benign disease [9–12]. However, few studies on using ARFI techniques to assess the outcome of RFA treatment in liver carcinoma have been reported [13]. In this prospective study, we aimed to investigate whether ARFI could be one alternative technique of CEUS to assess RFA outcomes in HCC.

## 2. Materials and Methods

### 2.1. Patients

The prospective study was carried out during May 2010 and December 2011. It was approved by the local research ethics committee and informed consent was obtained for each subjects. RFA was performed on 186 consecutive HCC patients; all of them had histopathological confirmation and did not have surgery indication or willingness. Patients included in this study had a tumor diameter equaling or less than 3 cm and the number of masses was ≤2. Exclusion criteria included: (1) patients could not hold their breathing; (2) the distance between lesion and skin greater than 8.0 cm; (3) lesions close to heart and big vessels such as aorta and inferior vena cava; (4) lesions close to dome of diaphragm (within a distance of approximately 5 cm) and lesions obscured by gas in intestine. 83 HCC masses in 72 patients satisfied the above inclusion and exclusion criteria. Of these patients, 61 patients showed single HCC mass, 8 patients had two HCC masses, and 3 patients had one recurred HCC mass after RFA. HCC patients with comorbidities of liver fibrosis (≤stage F3) due to hepatitis B or C and liver cirrhosis (stage F4) accounted for 83.3% (60/72) of the sample size.

### 2.2. Acoustic Radiation Force Impulse Method

The ultrasound system was Siemens Acuson S2000 with color Doppler ultrasonography and 4C1 convex array probe operating at the frequency of 2.0~4.0 MHz. This system was equipped with ARFI imaging and contrast enhanced-imaging pulse sequence. Conventional gray scale ultrasound was performed to show the lesions before and after the RFA. VTI was initiated with appropriate adjustments of sampling frame size, until the ablation lesion and parts of the surrounding liver parenchyma were covered. The lesion sites were divided into three categories based on their brightness, being high-echo (softer), iso-echo (similar echo-intensity as the surrounding liver parenchyma), and low-echo (harder). After VTI images acquisition and storage, VTQ technology was initiated which involves the selection of an anatomic region to be interrogated for elastic properties with the use of an ROI cursor by placing a “measuring box” of 10 mm long and 5 mm wide. The ROI was included entirely within the lesion, excluding all vessels and biliary structures. The shear waves were detected by sonographic detection pulses and the numeric values of the shear wave velocity (SWV, m/s) were calculated and displayed on the monitor. For liver parenchyma and pre-RFA measurement lesion, seven measurements were carried out on each lesion with sampling frame distributed evenly in the lesion. The highest value and lowest value were discarded; the average of the remaining 5 values was taken as the measurement for the lesion [14, 15]. For post-RFA measurement, at least 7 measurements were attempted and the sampling frame evenly covered the tumor. When measurements were out of the tolerable range of the system for shear wave velocity calculation, the shear wave velocity was displayed with “x.xx” [12, 13]. If the system displays “x.xx m/s”, according to the literature, a value of 9 m/s was used after the exclusion of cystic changes in the lesion [12]. Our previous experiences suggest that any post-RFA measurement with value lower than 9 m/s would suggest viable tissue, and therefore residue tumor [16].

### 2.3. Contrast-Enhanced Ultrasound

Ultrasound contrast agent (SonoVue, Bracco, Italy) was a freeze-dried powder formulation of sulfur hexafluoride phospholipid. Before use, 5 mL saline solution was mixed with the powder into a homogeneous suspension by oscillation. 2.4 mL of this contrast agent was injected through the elbow superficial vein by a rapid bolus followed by 5 mL saline flush, then a built-in timer started ultrasound scanning. With the entering of CEUS status, acoustic output power, focus and gain were adjusted, and the mechanical index was set to 0.05 using a low mechanical index gray-scale continuous real-time ultrasound scanning technology. Real-time continuous observation of the lesion contrast perfusion and echo intensity were carried out. If the arterial phase perfusion of contrast agent was required again for the ablation assessment, the second contrast injection was made 15 min later than the initial injection. Dynamic contrast-enhanced ultrasound movies and single-frame still images were recorded and then reviewed.

### 2.4. Contrast-Enhanced CT or MRI

**Figure 1:**
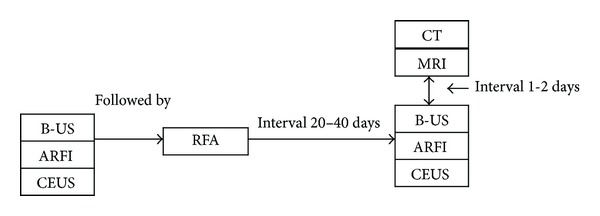
A flow diagram of the imaging and radiofrequency ablation (RFA) schedule.

Tissue surrounding the RF ablated surrounding may have the short term inflammatory edema. Contrast-enhanced CT or MRI evaluation of liver was performed approximately one month after the ablation so that inflammatory edema subdued (Figure 1). Siemens Somatom Sensation 64-slice spiral CT or GE Signa HD 1.5T magnetic resonance imaging system were used for examinations.

### 2.5. Radiofrequency Ablation Treatment

Patients were treated with RFA using the Cool-tip RF System (Radionics, Burlington, Massachusetts, USA) with 0–200 W of power and 480 kHz of frequency, and at cold cycling and RF pulse transmission mode. Ultrasound was performed to determine the suitable puncture track, followed by local anesthesia and RF electrode was advanced into the tumor interior. The intended ablation area covers the entire tumor and extended 1.0 cm beyond the border. Treatment started after confirming the correct location of the RF electrode. The treatment lasted 12 min each time.

### 2.6. RAF Efficacy Evaluation

Conventional ultrasound, ARFI, CEUS, contrast-enhanced CT, or MRI were read, respectively, by one experienced sonographer and one radiologist blinded to patient history, and the lesion size or ablation size measurement was performed. Lesion ablation measurement was performed at the same standard section for conventional B mode ultrasound, CEUS, enhanced CT, or MRI. For residual lesion assessment, if both the interior and peripheral parts of the primary tumor showed no enhancement, the post-treatment results of “complete ablation” was established. If radiologic evaluation showed the post-ablation lesion had interior or rim enhancement at arterial phase with portal phase subsiding, then the diagnosis of “partial residual tumor” was established.

Statistical analysis was performed using software SPSS (version 12.0 for Windows; SPSS, Chicago, Ill). Mann Whitney *U* test was used for comparison between the groups. One-way analysis of variance (ANOVA) and linear trend test was used in comparing SWV and ablation size in liver cirrhosis, liver fibrosis, and normal liver parenchyma subjects.

Reexamination of routine ultrasound, CEUS, ARFI, and contrast-enhanced CT or MRI was performed in about 20~30 days after RFA (Figure 1). Enhanced CT or MRI manifestations was regarded as reference standard for assessing residual tumor after RFA. Biopsy was performed for residual tumors when detected by post-RFA imaging.

## 3. Results

**Figure 2:**
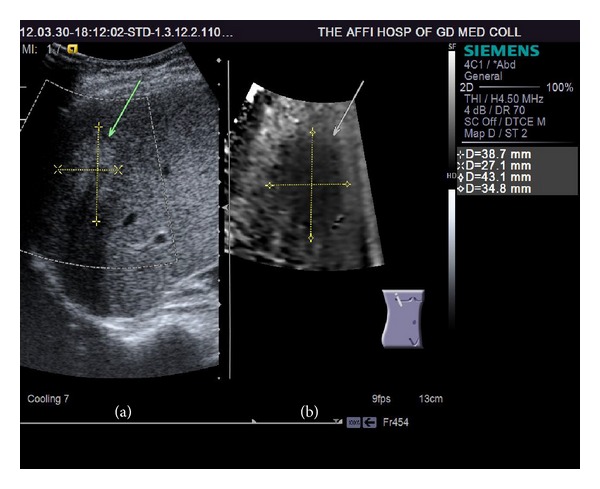
2D conventional US (a) and virtual touch tissue imaging (VTI, b). Tumor mass after RFA is remarkably darker (stiffer) than adjacent hepatic parenchyma on VTI. The ablated size of VTI (34.8 mm × 43.1 mm) is larger than that of 2D conventional US (27.1 mm × 38.7 mm).

**Figure 3:**
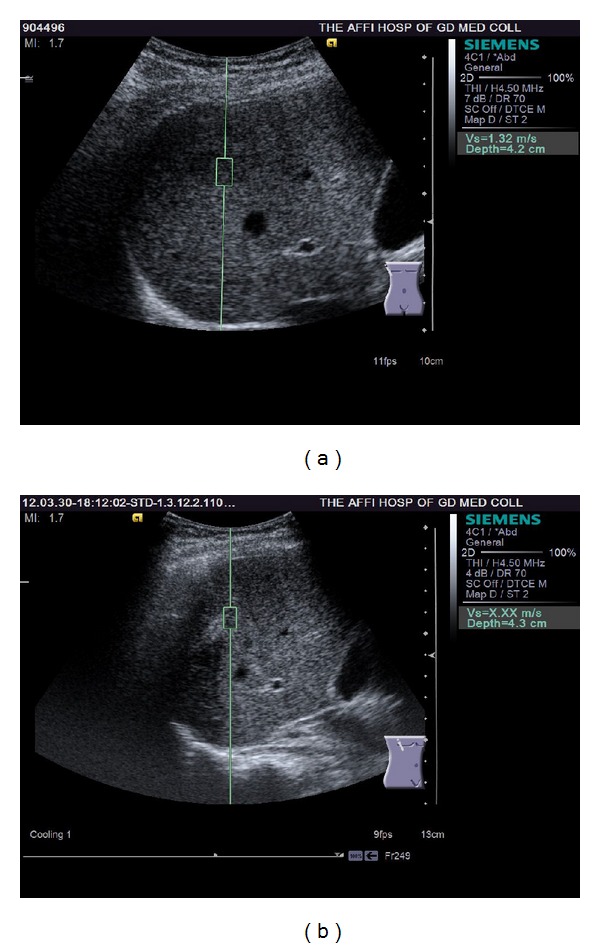
Virtual touch tissue quantification (VTQ) before (a) and after (b) RFA. Before RFA the shear wave velocity of this tumor is 1.32 m/s, while after RFA the shear wave velocity in the ablated area shows x.xx m/s (i.e. out of the range of the measurable values).

Prior to RFA procedure, on VTI images 25 tumors out of 83 (30.1%) were in the high-echo group, 17 tumors (20.5%) fell into the iso-echo group, and 41 tumors (49.4%) were in low-echo group. All tumors displayed low-echo after the RFA (Figures 2 and 3). The lesion size was 2.39 ± 0.47 mm with B mode US measurement, while was 3.03 ± 0.53 mm with VTI measurement (*P* < 0.05). Lesion size measurement from CEUS was 2.93 ± 0.51 mm (*P* > 0.05 versus VTI measurement). B mode measurement underestimated the lesion area (Figure 3), while ARFI-VTI measurement had good agreement with CEUS.

**Figure 4:**
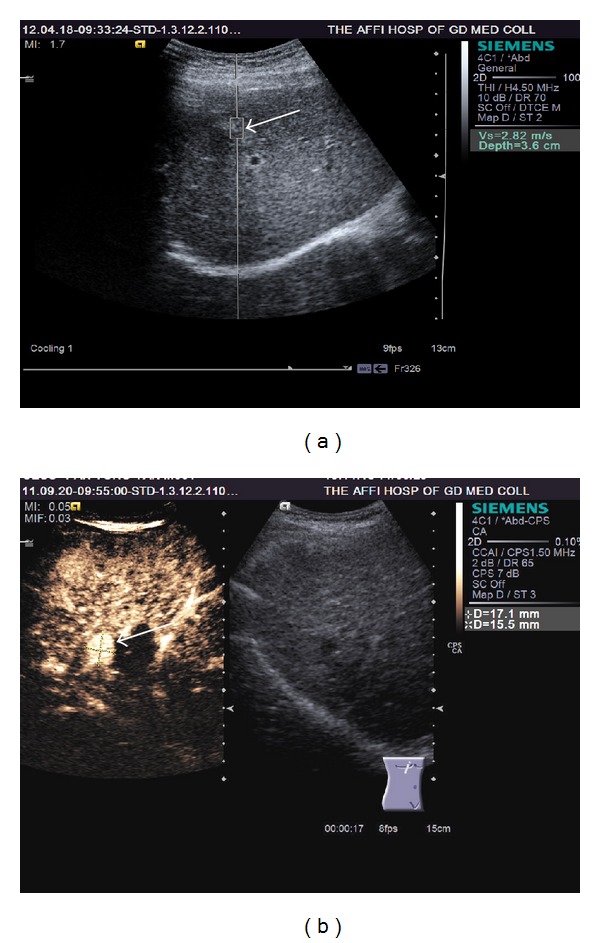
(a): Virtual touch tissue quantification (VTQ) in a HCC residual tumor area (arrow) after RFA with a shear wave velocity of 2.82 m/s. (b): CEUS image of the same residual tumor. Left part of lesion with residual tumor (arrow) shows enhancement after contrast injection whereas contrast perfusion cannot be observed in the completely ablated area. The border between residual tumor area and complete ablation area can be displayed clearly by CEUS.

Among the 83 tumor lesions, after RFA there were 14 lesions had residual tumors detected by CT or MRI (Table 1). VTI was not able to detect residual tumors. Out of the 14 residual tumor lesions, 13 were detected by CEUS (Figure 4), and CEUS missed one lesion with residual tumor. CEUS reported two false positive residual tumors which was negative on CT or MRI, and confirmed to be negative by biopsy. Compared with those had completed ablation, the 14 tumors with residual nodule had no difference in lesion size, both before and after RFA procedure, and also there was no tumor SWV difference before RFA procedure (Table 2). All the completely ablated nodules had a SWV demonstration of x.xx. While with those residual nodules, 6 (42.9%) had x.xx measurement, and 8 (57.1%) had measurable SWV (3.08 ± 0.59 m/s).

When the 83 tumors were divided into three groups based on whether the patients had normal parenchyma (*n* = 12, 14.5%), liver fibrosis (*n* = 44, 53.0%), or liver cirrhosis (*n* = 27, 35.5%), the liver parenchyma SWV showed a value of 1.52 ± 0.412 (m/s), 2.13 ± 0.42 (m/s), 2.71 ± 0.44 (m/s), respectively, representing a significant increasing trend (*P* < 0.05); while the ablation size showed a value of 4.17 ± 0.74 (cm), 4.16 ± 0.60 (cm), and 4.07 ± 0.50 (cm), respectively, representing a significant decreasing trend (*P* < 0.05). 9 lesions with residual tumor occurred in liver cirrhosis subjects (9/27, 33.3%), 5 lesions with residual tumor occurred in liver fibrosis subjects (5/44, 11.4%), while there was no residual tumor in the normal liver parenchyma subjects (0/12). Therefore there was an increased possibility of residual tumor associated with increasing liver parenchyma stiffness.

## 4. Discussion 

Local therapies, especially percutaneous ablation therapies, have gained increasing attention in treatment for liver cancer because of their advantages such as mini-invasiveness, easy manipulation, repeatability, and cost-effectiveness. RFA technique employs ultrasound, CT, or other imaging techniques to guide an electrode needle to be inserted into the interior of tumors, and it causes local lesions tissue temperature to arise, resulting in localized tumor lesion hyperthermia and coagulation necrosis [2]. Ultrasound-guided RFA is particularly widespread for HCC treatment [11]. A potential eradicative RFA should include the entire tumor plus a 5–10 mm peritumoral safety margin in an ideal sphere of necrosis, whereas an area of coagulation smaller than expected may lead to local recurrence [17]. In clinical practice, many factors (i.e., probe gauge, tip length, temperature achieved, heating duration, heat-sink effect of nearby blood vessels, and incomplete fusion of RFA lesions between prongs of expandable electrodes or surgical clips near the tumor) may alter this ideal geometrical shape producing an irregularly sized, distorted, or incomplete area of necrosis. It has been reported that conventional RFA devices with a single electrode or deployed electrode arrays with a thermal diameter of 3–4 cm provide a complete ablation rate of more than 90% for small tumors, but yield lower rates of 53–61% for medium-sized tumors (diameter 3.1–5 cm) and 20–45% for larger tumors (>5 cm) [17]. To correctly judge the location and extent of necrosis after RFA is a key evaluation for treatment effect, and it is directly related to prognosis of the patient.

Enhanced CT and MRI are often used as the reference diagnostic standard for post RF ablation efficacy evaluation. Enhanced CT and MRI determine whether RFA ablated completely the tumor or there are residual tumors based on whether the tumor is enhanced, that is, to use whether the contrast agent enters the tumor as a criterion. No enhancement means that the ablation is complete, with the pathological basis that when coagulation necrosis occurs there is no blood supply to the tumor, and therefore without contrast agent entering the tumor. By using ultrasound contrast agents and contrast specific imaging techniques, CEUS is able to depict the micro and macrocirculation in the liver and the treated lesion, thus allowing assessment of the treatment efficacy for HCC after percutaneous ablation therapy in a similar fashion with CECT or CEMRI [10, 18–21]. Different to CT or MRI contrast agents, ultrasound contrast agents do not diffuse into the intercellular space, and as such they reflect the blood supply of liver tumors by observing blood microcirculation characteristics. CEUS, similar to CECT or CEMRI, also follows the guideline of the modified Response Evaluation Criteria in Solid Tumor (mRECIST) [22]. In this guideline, viable HCC is defined as uptake of contrast agent in the arterial phase of CEUS; while complete response is defined as disappearance of any intratumoral arterial enhancement in HCC lesions [22, 23]. Researches showed CEUS in the assessment of RF ablation of liver cancer have the similar accuracy as contrast enhanced CT or MRI [24, 25].

VTI and VTQ are used to obtain tissue elasticity image and elasticity value, respectively. After RF ablation tissue stiffness in the necrotic areas is different from the surrounding normal tissues. By detecting the change of tissue hardness ARFI is used to determine the extent of inactivation of tumor. Among the 83 lesions, we carefully compared the 14 residual tumors with the 69 tumors had a complete ablation. The VTI display was all black, that is, overall hardness of the ablated zone being higher than the surrounding liver parenchyma. As the previous report by Kwon et al. [13], all the tumors appeared dark color after RFA treatment. This is likely due to that the RF ablated site was comprised of hard lesions that showed coagulative necrosis and fibrotic scarring [13]. This study shows that VTI imaging could estimate the tumor lesion area accurately, being consistent with the values from CEUS, and show a larger measurement than those obtained from the conventional B mode ultrasound. It has been previously reported that CEUS shows larger HCC size than conventional US measurement [26].

Our study suggested VTI could not distinguish between tumor residuals and complete ablation. This is consistent with results reported by Fahey et al. [27]. Kwon et al. [13] reported completely ablated lesion had VTQ value of x.xx. The 62/83 (74.7%) completely ablated lesions in our study agreed with Kwon et al's report, all showing x.xx measurement. With the 14 residual tumor lesions, 6 residual lesions also had a VTQ value of x.xx, another 8 lesions demonstrated measurable values. It is possible that the measurable SWV value may be partially due to the sampling frame of ROI being relatively large and may sample both residual tumor and necrotic tissues. Our results suggest that a post-RFA VTQ of measurable elasticity values indicates residual tumors, while a x.xx measurement cannot guarantee a complete ablation.

Our study demonstrated that liver cirrhosis was associated with decreased chance of a complete ablation of HCC. With the normal liver parenchyma, liver fibrosis, or cirrhosis, the ablation sizes were 4.17 ± 0.74 (cm), 4.16 ± 0.60 (cm), and 4.07 ± 0.50 (cm), respectively, representing a significant decreasing trend. 9 residual tumors occurred in liver cirrhosis subjects (9/27, 33.3%), 5 residual tumors occurred in liver fibrosis subjects (5/44, 11.4%), while there was no residual tumor in the normal liver parenchyma subjects (0/12). In a recent study by Kang et al. [17], the correlation between RFA extent and hepatic parenchymal SWV was evaluated. It was shown that a highly significant negative correlation between parenchymal mean SWV and RFA extent. The results showed the higher the parenchymal SWV, which indicates hepatic fibrosis, the smaller the RFA extent, which indicates decreasing RFA efficacy. Therefore the parenchymal SWV measured by ARFI is an important parameter in the prediction of RFA treatment extent. Kang et al. suggested if the peritumoral SWV is higher than the cut-off value 2.41, multiple session of RFA may be needed to ablate the tumor with free safe margin [17].

There are a number of limitations for the current study. The major weak point of this study is that the measured stiffness on ARFI imaging was not correlated with real stiffness determined by histopathological examination. Patients with limitations of ARFI application were excluded from the study, such as those tumors located too deep (>8 cm). Additionally, we did not link the ARFI or CEUS results to the survival data. Out of the 14 residual tumor lesions, CEUS missed one lesion with residual tumor and reported two false positive residual tumors. These results suggested CEUS was slightly inferior in assessing residual tumor after RFA than contrast enhanced CT/MRI. This result has been recently reported by Zheng et al. [18]. Hyperenhancement in the arterial phase of CEUS lasts only few seconds so CEUS has no enough time to scrutinize the whole lesion. As a consequence, CEUS may miss some residual tumor tissue that declines from hyper to iso or hypoenhancement [18].

In conclusion, our study suggested that while VTI technique shows accurate lesion size estimation, it cannot reliably demonstrate residual tumors post RFA. All fully ablated tumors have VTQ measurement of x.xx, but VTQ results of x.xx in the ablated lesion cannot exclude residual tumor. ARFI cannot replace CEUS or CT/MRI for residual tumor assessment. Our study further confirms liver cirrhosis is associated with decreased RFA efficacy for HCC.

## Figures and Tables

**Table 1:** ARFI measurements of 14 residual tumors.

Sex	Age	Comorbidity	Tumor size*	Tumor size*	Tumor size*	SWV**	RFA size*
			B-mode	VTI	CEUS	after RFA	VTI
M	62	Cirrhosis	2.72	2.89	2.81	x.xx	3.78
M	72	Fibrosis	1.91	2.22	2	x.xx	3.3
M	50	Fibrosis	2.22	2.51	2.5	3.15	3.14
M	72	Cirrhosis	1.58	3.02	2.82	3.97	3.66
M	55	Cirrhosis	2.5	3.52	3.33	3.78	4.24
F	74	Fibrosis	1.98	3	3	2.57	3.63
M	64	Cirrhosis	2.31	3	3	x.xx	3.7
M	73	Fibrosis	2.6	3.13	3	3.12	4.12
M	65	Cirrhosis	3	3.82	3.71	2.47	4.58
F	71	Fibrosis	3	3.61	3.46	2.38	4.26
M	60	Cirrhosis	1.72	2	2	3.21	3.69
M	61	Cirrhosis	2.38	2.92	2.87	x.xx	4.51
M	59	Cirrhosis	3	3.65	3.54	x.xx	4.23
F	54	Cirrhosis	2.6	3.14	3	x.xx	4.6

M: male; F: female; ARFI: acoustic radiation force impulse; HCC: hepatocellular carcinoma; SWV: shear wave velocity; RFA: radiofrequency ablation; VTI: virtual touch tissue imaging; *unit in cm; **unit in m/sec.

**Table 2:** The ARFI results comparison between the 14 residual tumors and 69 complete ablated sites.

	*n*	VTI before RFA	VTI after RFA	Tumors site before RFA	Liver parenchyma tissue
		size (cm)	size (cm)	SWV (m/s)	SWV (m/s)
Completely ablated tumors	69	2.93 ± 0.56^§^	4.19 ± 0.63^§^	2.45 ± 0.54^§^	2.01 ± 0.55*
Lesions with residual tumor	14	3.03 ± 0.53^§^	3.91 ± 0.46^§^	2.31 ± 0.57^§^	2.65 ± 0.45*

^§^Indicates no significant difference between the two groups (*P* > 0.05).

*Indicates significant difference between the two groups (*P* < 0.05).
